# Genetic Architecture and Candidate Genes Identified for Follicle Number in Chicken

**DOI:** 10.1038/s41598-017-16557-1

**Published:** 2017-11-27

**Authors:** Manman Shen, Hongyan Sun, Liang Qu, Meng Ma, Taocun Dou, Jian Lu, Jun Guo, Yuping Hu, Xingguo Wang, Yongfeng Li, Kehua Wang, Ning Yang

**Affiliations:** 1 Jiangsu Institute of Poultry Science, Chinese Academy of Agricultural Science, Yangzhou, China; 2 College of Animal Science and Technology, Yangzhou University, Yangzhou, China; 3 National Engineering Laboratory for Animal Breeding and MOA Key Laboratory of Animal Genetics and Breeding, College of Animal Science and Technology, China Agricultural University, Beijing, China

## Abstract

Follicular development has a major impact on reproductive performance. Most previous researchers focused on molecular mechanisms of follicular development. The genetic architecture underlying the number of follicle, however, has yet not to be thoroughly defined in chicken. Here we report a genome-wide association study for the genetic architecture determining the numbers of follicles in a large F_2_ resource population. The results showed heritability were low to moderate (0.05–0.28) for number of pre-ovulatory follicles (POF), small yellow follicles (SYF) and atresia follicles (AF). The highly significant SNPs associated with SYF were mainly located on GGA17 and GGA28. Only four significant SNPs were identified for POF on GGA1. The variance partitioned across chromosomes and chromosome lengths had a linear relationship for SYF (R^2^ = 0.58). The enriched genes created by the closest correspondent significant SNPs were found to be involved in biological pathways related to cell proliferation, cell cycle and cell survival. Two promising candidate genes, AMH and RGS3, were suggested to be prognostic biomarkers for SYF. In conclusion, this study offers the first evidence of genetic variance and positional candidate genes which influence the number of SYF in chicken. These identified informative SNPs may facilitate selection for an improved reproductive performance of laying hens.

## Introduction

The reproductive performance of hens is an indicator of egg production, which occurs following a strict follicular hierarchy resulting from a process of orderly growth which begins with recruitment, followed by selection and, lastly, ovulation. During reproduction period, the majority of small yellow follicles (<8 mm in diameter) become atretic and are reabsorbed, with only 5% of follicles further developing into pre-ovulatory follicles (8–40 mm in diameter). Typically, a single SYF is selected daily to join the hierarchy POF for ovulation during peak laying periods^1,2^. Therefore, thoroughly understanding the process of follicular development and its mechanisms could aid in significantly improving egg production.

In chicken, most previous studies focused on the molecular mechanisms of follicular growth *in vitro* ^3^, which were associated with numerous candidate genes including FSHR, ESR, PRL and PRLR^3,4^. Follicle numbers were also a key determinant of laying performance and were affected by breed^5^, age^6^, and nutrition^7^. For instance, researchers found a big difference in follicle numbers between old laying hens (e.g. >1 year of age) and laying hens in peak stage^8^. Recently, a couple of genome-wide association studies (GWAS) conducted to study follicle numbers revealed that they are associated with menopausal age^9^ in humans, and ovarian function and fertility in cattle^10^. However, no genetic variants influencing follicular numbers have been reported in chicken thus far, especially using GWAS to investigate them directly.

In the present study, we conducted a GWAS to discover genomic regions explaining variations in follicle numbers of White Leghorn and a Chinese indigenous chicken, the Dongxiang Blue-shelled Chicken, using the 600 K Affymetrix Axiom Chicken Genotyping Array. The objective of this study was to investigate the genetic architecture underlying follicles and identify genetic markers of follicle numbers which would employ molecular marker-assisted selection on laying performance.

## Results

### Phenotypic measurements and genetic parameters

The overall means, standard errors, coefficients of variation, and heritability are illustrated in Table 1. The mean of the number of POF, SYF, and AF was 3.84, 16.36, and 4.91, respectively, with small standard errors (Table 1). The numbers of the three types of follicles had large coefficients of variation that ranged from 30.13% to 55.97% (Table 1). SYF had a moderate heritability (0.28), while POF and AF had low heritability of 0.13 and 0.05, respectively (Table 1). Both genetic and phenotypic correlation between each two of the three types of follicles were positive under bivariate GCTA analyses (Table S1).

**Table 1:** Phenotype data and genetic parameters.

Trait	Mean ± SE	Max	Min	CV (%)	h^2^ (SE)
POF	3.84 ± 0.03	13	0	30.13	0.13 (0.03)
SYF	16.36 ± 0.20	63	0	46.58	0.28 (0.04)
AF	4.91 ± 0.09	25	0	55.97	0.05 (0.04)

Note: POF, pre-ovulatory follicle; SYF, small yellow follicle; AF, atresia follicle; CV, coefficient of variation; SEM, standard error.

### Genome-wide association study (GWAS)

**Figure 1:**
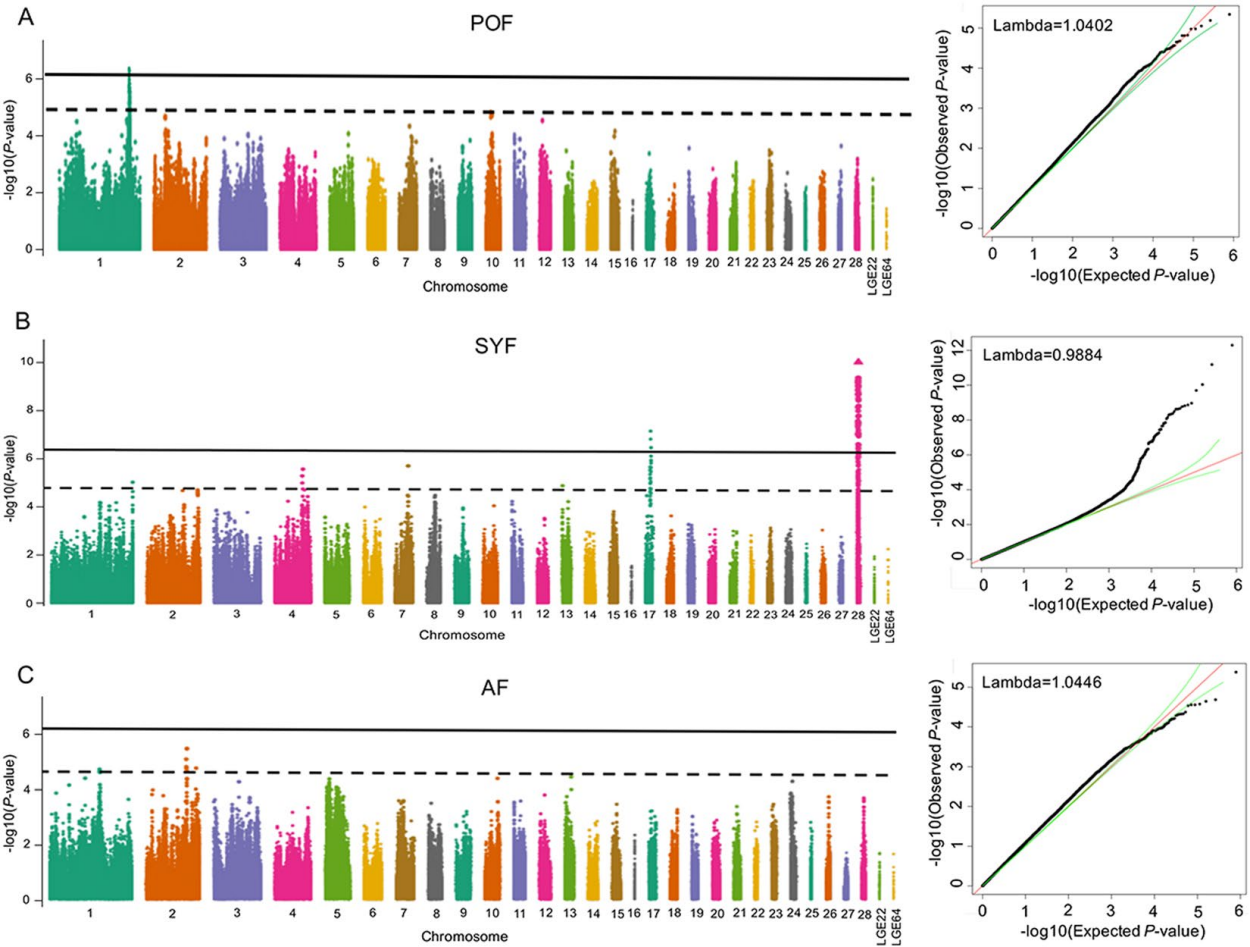
Manhattan plot (left) displaying the association of all SNPs with preovulation follicle (POF; (**A**), small yellow follicle (SYF; (**B**), and atresia follicle (AF; (**C**) from GEMMA GWAS. The x-axis indicates the position of SNPs on each chromosome. The y-axis is the −log10 (observed P values) for genome-wide SNPs. The horizontal solid black and dash black line indicate the genome-wide significant (8.43 × 10^−7^) and suggestive significant (1.69 × 10^−5^) association thresholds, respectively. The quantile-quantile plots (right) shows the observed distribution of P values against the expected P values under the null hypothesis of no association.

By analyzing a total of 1456 hens, we identified 55 genome-wide significant SNPs and 55 suggestive significant SNPs associated with SYF via univariate analyses in GEMMA (Table S2). Four significant SNPs on GGA1 passed the threshold of strong association (8.43 × 10^−7^) for POF (Table S3). However, no SNPs displayed significant genome-wide association with AF. The Manhattan plot (Fig. 1 left) shows the P values for all SNPs affecting each trait, across all chromosomes, while the quantile-quantile (QQ) plot (Fig. 1 right) displays significant deviations of observed P values from those expected. The deflation factor (*λ*) was estimated at 1.0402, 0.9884, and 1.0446 for POF, SYF, and AF, respectively, indicating a good consistency between the observed and expected P values (Fig. 1 right).

**Figure 2:**
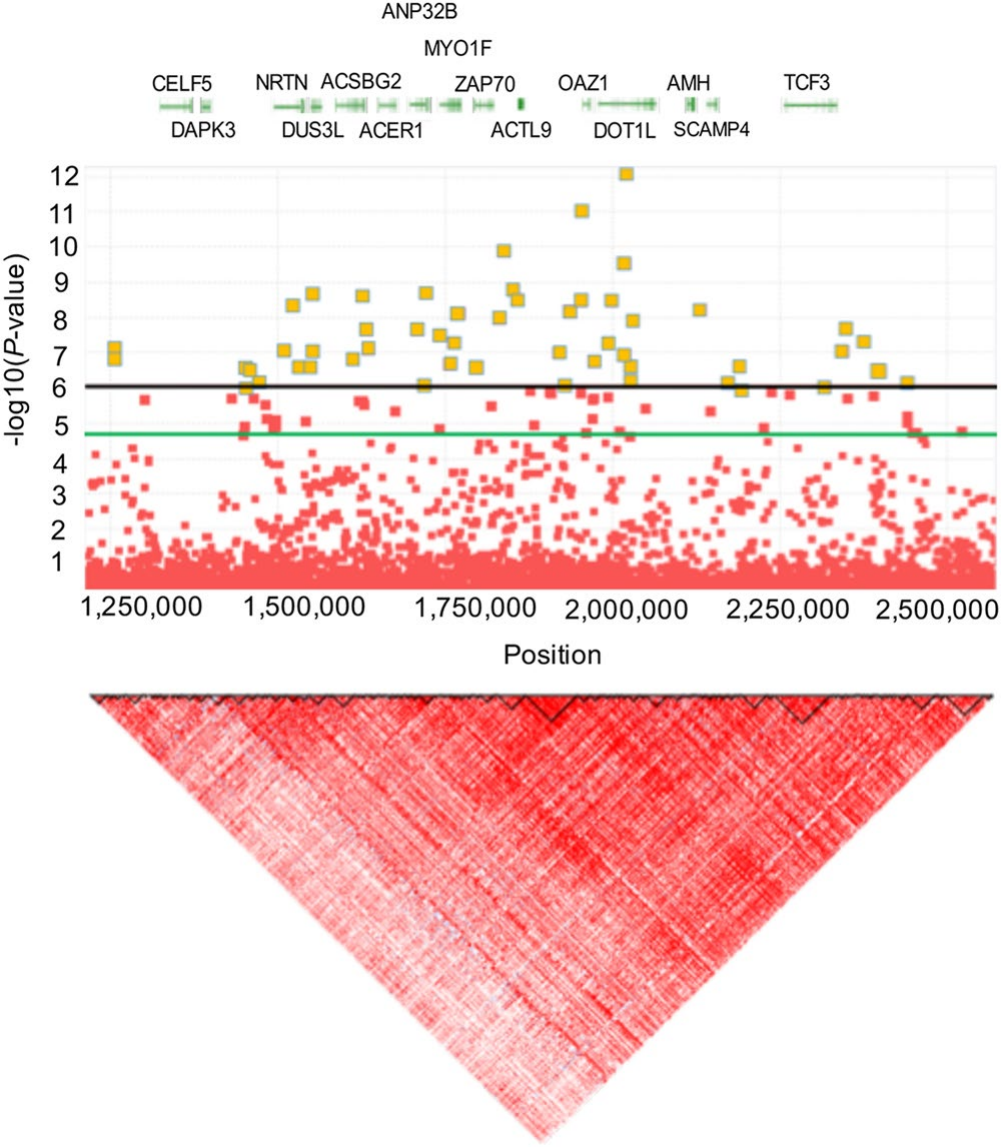
Linkage disequilibrium plots of significantly associated genetic and functional regions. D’ ≥ 0.8 indicates the strong LD block. The horizontal solid black and blue line indicate the genome-wide significant (8.43 × 10^−7^) and suggestive significant (1.69 × 10^−5^) association thresholds, respectively.

The genome-wide significant association SNPs for SYF were located in a 1.25 Mb region spanning from 1.25 Mb to 2.5 Mb on chromosome GGA28. The most significant SNPs associated with SYF on chromosome GGA28 spanned from 1.4 to 2.5 Mb and showed substantial linkage disequilibrium (LD; Fig. 2). A total of 64 small scale blocks were observed in this region. The identified SNPs with the strongest association with SYF were rs317889060 and rs316038837 (Table S2).

When analyzing the genomic selection, the Bayes B method in GenSel used to identify significant loci associated with SYF generated similar results. The significant variation threshold for the Bayes B method was selected as 0.5% for each window. The highest effect of SNPs on the SYF trait was listed in Table S4, for each significant window obtained via GenSel. A total of 42 SNPs were identified as significantly associated with the SYF trait. From these, twenty-eight significant SNPs were also detected in GEMMA (Table 2), indicating a high correlation (>50% common loci) between the two methods.

**Table 2:** Results of multi-marker (GEMMA) and Bayesian B (GenSel) genome-wide association analysis for small yellow follicle.

Chr	SNP	Position	GEMMA				GenSel		
			Minor/Major allele	beta	s.e	P vaule	Window (SNPs)	%GV	Model freq.
28	rs316444293	2342019	C/A	3.29E-01	5.77E-02	1.39E-08	125	0.63	0.0048
28	rs316038837	1947008	T/C	4.02E-01	5.79E-02	6.57E-12	148	0.67	0.0063
28	rs16210881	1829370	G/A	3.62E-01	5.53E-02	9.27E-11	322	0.73	0.0042
28	rs313648246	2010929	A/T	2.90E-01	4.52E-02	2.01E-10	255	0.57	0.0053
28	rs316105069	2123244	A/G	3.66E-01	6.19E-02	4.28E-09	156	0.69	0.0037
28	rs318236639	1929393	A/G	2.91E-01	4.96E-02	4.80E-09	169	1.23	0.0056
28	rs314802132	1542976	C/A	3.09E-01	5.08E-02	1.53E-09	218	0.54	0.0053
28	rs313479236	1821752	C/T	2.99E-01	5.16E-02	7.16E-09	129	0.58	0.0049
28	rs313972624	1732609	C/A	2.80E-01	5.00E-02	2.22E-08	216	0.78	0.0068
28	rs317337799	1754154	C/T	2.51E-01	4.55E-02	3.63E-08	226	0.53	0.0145
28	rs316358363	1626264	A/G	3.15E-01	5.77E-02	5.45E-08	175	0.55	0.0052
28	rs14305824	2179908	A/G	2.87E-01	5.49E-02	1.73E-07	263	0.65	0.0063
28	rs316367068	1524526	A/G	2.71E-01	5.19E-02	1.76E-07	145	0.63	0.0038
28	rs312811524	1786822	C/G	2.38E-01	4.55E-02	1.76E-07	128	0.74	0.0043
28	rs13663720	1788269	T/C	3.19E-01	6.12E-02	1.85E-07	312	0.66	0.0049
28	rs312586874	1449431	A/G	2.65E-01	5.10E-02	1.96E-07	215	0.56	0.0053
28	rs16211274	1463378	A/G	2.46E-01	4.89E-02	4.70E-07	179	1.26	0.0046
28	rs318099911	2163700	C/T	3.06E-01	6.09E-02	4.98E-07	268	0.54	0.0053
28	rs316574537	2432874	T/C	2.86E-01	5.68E-02	4.98E-07	219	0.52	0.0059
28	rs314231916	1709862	A/G	2.29E-01	4.56E-02	5.52E-07	316	0.69	0.0068
28	rs314907214	1443055	G/A	2.49E-01	5.02E-02	6.98E-07	326	0.53	0.0125
17	rs312873273	1366852	G/C	3.02E-01	5.33E-02	2.20E-08	275	0.55	0.0042
28	rs16211139	1602011	G/C	2.69E-01	5.05E-02	1.02E-07	163	0.75	0.0053
28	rs315697880	1623888	C/T	3.28E-01	5.78E-02	1.57E-08	155	0.53	0.0048
28	rs14306530	1499327	T/C	3.24E-01	5.97E-02	5.73E-08	188	0.64	0.0053
28	rs16211213	1541935	A/G	3.15E-01	5.81E-02	6.36E-08	322	0.66	0.0039
28	rs314643774	1910877	G/A	2.46E-01	4.55E-02	7.03E-08	255	0.76	0.0033
28	rs316413369	1537133	T/C	2.40E-01	4.56E-02	1.76E-07	279	0.66	0.0056

### Annotation of significant SNPs and *in silico* pathway analysis

The four detected SNPs for POF was mapped to DCLK1, NBEA, and nearby SMAD9. The detailed annotations of the 28 SNPs significantly associated with the SYF trait, identified using both methods, were listed in Table 2. Six intergenic variant SNPs were associated with the SYF trait (Table 3). In addition, thirteen SNPs were located up- or down-stream of genes including TCF3, SCAMP4, OAZ1, DUS3L, ZAP70, ACSBG2, REXO1, NRTN, NMRK2, ABHD17A, ANP32B, and SNORD37 (Table 3). Two SNPs were mapped to non-coding transcript region and 1 SNP was synonymous variant. Also of note, six SNPs were encompassed in the introns of the DOT1L, MYO1F, ADAMTS10, SH3GL1, and RGS3 genes (Table 3).

**Table 3:** Positional candidate genes categorized by function for common detected SNPs from two methods.

Chr	SNP	Position	Candidate genes/Nearest genes	Location	Description
28	rs316444293	2342019	TCF3	Upstream 1027 bp	Transcription factor 3
28	rs316038837	1947008	Upstream 14 kb of AMH	Intergenic variant	Anti-Mullerian hormone
28	rs16210881	1829370	LOC101748229	Intron variant	Nuclear factor interleukin-3-regulated protein-like
28	rs313648246	2010929	DOT1L	Intron variant	DOT1 like histone lysine methyltransferase
28	rs316105069	2123244	SCAMP4	Downstream 999 bp	Secretory carrier membrane protein 4
28	rs318236639	1929393	OAZ1	Downstream 1549 bp	Ornithine decarboxylase antizyme 1
28	rs314802132	1542976	DUS3L	Upstream 4541 bp	Dihydrouridine synthase 3 like
28	rs313479236	1821752	ZAP70	Downstream 4223 bp	Zeta-chain (TCR) associated protein kinase 70 kDa
28	rs313972624	1732609	MYO1F	Intron variant	Myosin IF
28	rs317337799	1754154	ADAMTS10	Intron variant	ADAM metallopeptiddase with thrombospondin type 1 motif, 10
28	rs316358363	1626264	ACSBG2	Upstream 4223 bp	Acyl-CoA synthetase bubblegum family member 2
28	rs14305824	2179908	REXO1	Upstream 2997 bp	RNA exonuclease 1 homolog
28	rs316367068	1524526	NRTN	Upstream 4113 bp	Neurturin
28	rs312811524	1786822	Downstream 40 kb of ADAMTS10	Intergenic variant	ADAM metallopeptiddase with thrombospondin type 1 motif, 10
28	rs13663720	1788269	Upstream 7 kb of ANP32B	Intergenic variant	Acidic (leucine-rich) nuclear phosphoprotein 32 family, member B
28	rs312586874	1449431	NMRK2	Downstream 2995 bp	Nicotinamide riboside kinase 2
28	rs16211274	1463378	NMRK2	Downstream 4803 bp	Nicotinamide riboside kinase 2
28	rs318099911	2163700	ABHD17A	Upstream 4844 bp	Abhydrolase domain containing 17 A
28	rs316574537	2432874	SH3GL1	Intron variant	SH3 domain containing GRB2 like 1, endophilin A2
28	rs314231916	1709862	ANP32B	Upstream 2156 bp	Acidic (leucine-rich) nuclear phosphoprotein 32 family, member B
28	rs314907214	1443055	SNORD37	Downstream 694 bp	Small nucleolar RNA SNORD37
17	rs312873273	1366852	RGS3	Intron variant	Regulator of G-protein signaling 3
28	rs16211139	1602011	Upstream 15 kb of DUS3L	Intergenic variant	Dihydrouridine synthase 3 like
28	rs314643774	1910877	Upstream 15 kb of OAZ1	Intergenic variant	Ornithine decarboxylase antizyme 1
28	rs315697880	1623888	Downstream 5 kb of DUS3L	Intergenic variant	Dihydrouridine synthase 3 like
28	rs14306530	1499327	FUT6	Synonymous variant	Fucosyltransferase 6
28	rs16211213	1541935	—	Non-coding transcript variant	Novel lincRNA
28	rs316413369	1537133	—	Non-coding transcript variant	Novel lincRNA

**Figure 3:**
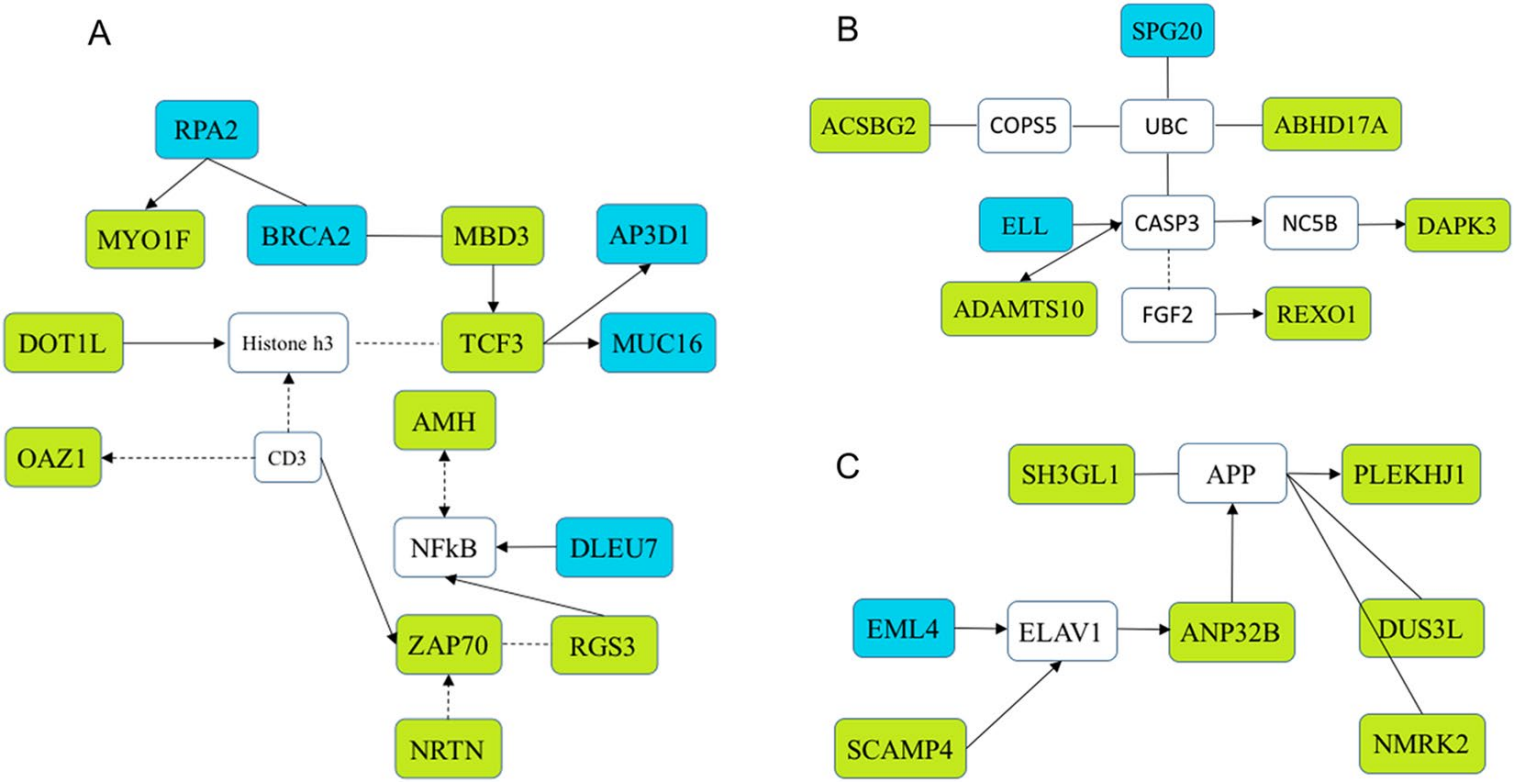
Network of interactions between GWAS positional candidate genes. The network shows interactions between the candidate genes enriched for significantly associated SNPs. (**A**) Tissue morphology, organismal injury and abnormalities. (**B**) Cell death and survival, organismal injury and abnormalities, cellular development. (**C**) Tissue morphology, cellular development, cell cycle. Green box indicates the detected candidate genes in current study, while blue box represents the genes identified from previous studies. The solid arrow line indicates direct relationship while the dash arrow line means indirect relationship.

To explore the potential functional implications of the detected SNPs and identify the biological pathways and processes that these SNPs affect, the *in silico* pathway analysis and the Cytoscape method were used (Table 4). The significant SNPs were marked abundantly in genes participating in cell development, survival, differentiation, and apoptosis, which is in line with our expectations. The three identified clustered gene networks were involved in general molecular and cellular processes such as cell cycle, cell survival, cell development, and organismal injury and abnormalities (Fig. 3). The first network is composed of genes with transcriptional control (MBD3, TCF3, DOT1L) and the AMH, ZAP70, RGS3, and OAZ1 enzymes. The second informative network is formed of network interacting molecules, including many enzymes such as ACSBG2, ADAMTS10, ABHD17A, DAPK3, and REXO3. The third cluster contains genes involved in cellular development and cell cycle, such as SH3GL1, SCAMP4, ANP32B, DUS3L, and NMRK2 *etc*.

**Tablee 4:** Functions enriched in the significant SNPs data set as identified *in silico* pathway analysis.

Functions	P value	Genes	Number of detected SNPs
Tissue morphology, cancer, organismal injury and abnormalities	6.05 × 10^−4^	RPA2, MYO1F, BRCA2, MBD3, AP3D1, DOT1L, Histone h3, TCF3, MUC16, OAZ1, CD3, AMH, NFkB, DLEU7, RGS3, ZAP70, NRTN	10
Cell Death and Survival, Organismal Injury and Abnormalities, Cellular Development	2.48 × 10^−4^	SPG20, UBC, ABHD17A, COPS5, ACSBG2, ELL, CASP3, NC5B, DAPK3, ADAMTS10, FGF2, REXO1	8
Tissue Morphology, Cellular Development, Cell Cycle	1.95 × 10^−3^	SH3GL1, APP, PLEKHJ1, EML4, ELAV1, ANP32B, DUS3L, NMRK2, CAMP4	5

### Features of allelic contribution

The contribution to SYF manifestation, as well as the genotype-effect of the more prominent SNPs in Table 5, is that these SNPs have a strong impact (2.46–5.5%) on the phenotypic variation of trait. Notably, rs3160388837 is the most significant SNPs on GGA28, located up-stream of AMH, exerting a 5.5% contribution to the phenotypic variation of SYF. According to the GWAS data, the TT genotype is linked with a higher incidence of SYF, whereas CC is the risk genotype for low numbers of SYF (Table 5). Another particularly interesting SNP is rs312873273, located on chromosome GGA17, which explains 2.4% of variation in the SYF trait.

**Table 5:** SNP and genotype effects of the two significant SNPs for small yellow follicle number.

Chr	SNP	MAF	CPV (%)	Phenotype (Genotype)		
28	rs316038837	0.176	5.50	15.46^C^ (CC)	17.95^B^ (TC)	23.00^A^ (TT)
17	rs312873273	0.213	2.46	14.95^B^ (CC)	17.91^A^ (CG)	16.95^A^ (GG)

Note: Chr, chromosome; MAF, minor allele frequency; CPV, contributions to phenotypic variance. The phenotypic data with different capital letters in same row means extremely significant difference.

### Genetic variation partition

**Figure 4:**
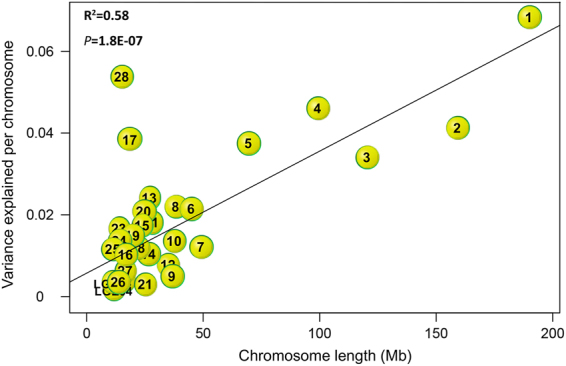
Scatterplot showing the genome partitioning for small yellow follicle (SYF). Solid lines are shown for linear regressions, which were significant (R^2^ = 0.58; P = 1.8e-7). The characters in the circles are the chromosome numbers.

The total genetic variance was partitioned onto individual chromosome, to dissect the genetic architecture of follicle development, by simultaneously fitting the genetic relationship matrix (GRM) of all the chromosomes. Only the SYF trait showed significant effects of individual chromosome on genetic variation (Fig. 4). Chromosome GGA28 and GGA17 explained the majority of the variation in SYF numbers, accounting for 5.7% and 3.9% of variance, respectively, which indicates different contributions of particular chromosomes to the SYF trait. Moreover, we observed a strong significant positive linear relationship (P = 1.8e-7, R^2^ = 0.58) between the estimated variance explained by each chromosome and the relative length of the chromosomes for SYF (Fig. 4).

## Discussion

The aims of this study were to identify the genetic architecture associated with the numbers of different types of follicles and explore the positional candidate genes for those traits using a novel F_2_ population and the 600 K SNP panel. To date, this is the first GWA study of follicle numbers in birds.

We used the commercial standard line White Leghorn and Chinese indigenous Dongxiang Blue-shelled Chicken to generate an F_2_ population, creating a unique opportunity to maximize the differences in traits, while increasing the power to identify SNPs for respective traits. Moreover, an extremely large population was used in this GWA study, as well as a higher density (600 K) SNP array, to increase the accuracy and reliability of the obtained data. Different types of follicles were selected for this GWA study, deepening our understanding of the genetic architecture underlying follicular development, from recruitment until ovulation.

Compared to a previous study of commercial hens^11^, the mean number of POF in the current study was slightly smaller, which may have been caused by the different breeds. In this study, identified heritability were low (0.13) for POF, which could be due to unavailability, using the clutch status of hens, once follicles in the hierarchy are selected for ovulation^12^. The extremely low heritability (0.05) of AF suggests that unknown internal and external factors may have affected this measurement. More precise methods and control mechanism to investigate POF and AF should be explored in a subsequent study. Heritability for SYF was moderate (0.28), which was similar to previously reported heritability of follicle numbers in cattle^10^. The heritability of SYF was higher than the ovary weight and POF weights in previous estimates^13^, but similar to the heritability of egg numbers^14^.

To accurately identify the potential loci affecting the number of chicken follicles, we used two different software systems –GEMMA and GenSel– to explore the observed associations for each trait. Very few SNPs associated with either POF or AF were identified using both software packages. However, a relatively high, consistent number of SNPs was identified genome-wide to significantly associate with SYF, using the two methods of analysis. Of the 55 (50.91%) most significant SNPs identified by GEMMA, 28 SNPs were also amongst the 42 (66.67%) most significant SNPs by GenSel. Both results showed evidence for association on chromosome GGA28 and GGA17 as multiple significant SNPs were located on these two chromosomes. Although most of the significant SNPs associated with SYF obtained in the current study were different from previous results of ovary weight and egg number^13,14^, the predicted positional candidate genes partially agree with previous reports^13,14^.

Two SNPs, rs16211213 and rs316413369, fall into non-coding regions of the genome, inferring that the function of these variations is the control of gene expression instead of variations in the gene product itself. The genes located near the significant SNPs were compiled as gene lists to perform an unbiased pathway analysis and build gene networks. All three resulting networks were tightly related to several of the genes (molecules) involved in cell development, cell cycle, cell death and survival. For example, MYO1F present in the first cluster network (Fig. 3A) has an ability to guide immune cell motility^15^; OAZ1 could regulate intracellular polyamine levels^16^; MBD3 is an essential requirement for pluripotency of embryonic stem cells^17^; DOT1L is over-expression in ovarian cancer, driving cell cycle progression^18^. Follicle growth results from a process of cell survival, differentiation, proliferation and apoptosis. Based on the present findings, it is possible to speculate that the genes within these networks play a key role in the genetic determination of SYF.

Other most promising candidate genes for SYF were AMH and RGS3. AMH, a glycoprotein, is a member of the TGF- β family, involved in tissue growth, cell differentiation and follicle development. Moreover, previous studies demonstrate that this gene is the essential in determining the number of primordial follicle^19^. Plasma AMH level is one of the criteria used to diagnose polycystic ovary syndrome (PCOS)^20^, which results in an anovulation, and subsequent infertility, in women of reproductive age. Besides, AMH is an excellent marker of ovarian reserve, as a decrease in AMH level is typically accompanied by ovarian senescence^21,22^. Also, AMH can inhibit the process of primordial follicle recruitment and follicle development^22^.

In this GWA study, we identified the significant SNP rs316038837 associated with the SYF trait, which is 14 kb up-stream AMH. In chicken, the expression level of AMH is strongly related to follicular growth, especially for small follicles^23^. Previous studies have demonstrated that AMH expression level in SYF, together with other genes such as BMP6, FSH, BMP15, played an essential role in follicle development^23–25^. Also, it was discovered that broiler ovaries had a higher AMH expression level than the standard commercial layer ovaries^5^, suggesting the inhibitory action of AMH in chicken follicle selection. The present GWAS results, along with the crucial role of AMH in chicken follicle development, indicate AMH might be an excellent candidate gene for SYF numbers and could have a similar role in mammalian ovarian folliculogenesis.

Another interesting positional candidate gene, identified in the present study, is RGS3 (rs312873273 associated with SYF) on chromosome GGA17. RGS3 encodes for a regulator of G-protein signaling^26^. Previous studies showed that RGS3 had negative regulatory functions on FHSR and LHR signaling in rats^27^. In human, this gene is involved in GnRH responsiveness in granular cells^28^. Moreover, many research studies on chicken showed the important roles of FSHR, FSH, and GnRH in follicular growth: the expression level of FSHR is tightly related to the number of pre-hierarchy follicles; FSH is critical to the recruitment of follicles; GnRH has an established role on the FSH synthesis^1^. The aforementioned information, leads to concluding that RGS3 might be an essential factor in follicular growth and have a potential inhibitory effect on follicle number.

Furthermore, the gene network analysis showed that the AMH and RGS3 genes can active NFκB (Fig. 3). NFκB is a transcription regulator, which can be induced by various stimuli, like AMH. The NFκB pathway may be involved in the basic control of ovary proliferation, apoptosis, and hormone release^29^. In swine, the NFκB signaling cascade is a mediator of FSH action which regulates AMH expression in ovarian cells^30^. In humans, AMH had an inverse relationship with breast tumor development and growth^31–34^. Based on the current results and previous studies, it is reasonable to speculate that AMH and RGS3 can regulate follicle development and growth *via* activation of the NFκB signaling pathway.

In summary, we have identified the first genetic variants influencing normal variation in the numbers of the different types of chicken follicles, especially SYF. The present GWAS results suggest that genetic variants of AMH (rs316038837) and RSG3 (rs312873273) genes could be used as prognostic biomarkers for SYF development. However, it will be important to further evaluate the role of these regions in follicular development. Since follicular development is strongly related to egg production, it will be interesting to examine whether variations in these genomic regions relates to reproductive performance. Also, these identified informative SNPs may facilitate selection for an improved productive performance of chicken.

## Methods

### Ethics statement

All animal care, slaughtered and experimental procedures were approved by the Institutional Animal Care and Institutional Ethic Committee of Jiangsu Institute of Poultry Science. All methods were performed in accordance with guidelines of the Jiangsu Institute of Poultry Science.

### Resource population

In brief, six males from each of White Leghorn (WL) and a Chinese indigenous chicken, the Dongxiang Blue-shelled Chicken (DX), were mated to 133 and 80 females from DX and WL, respectively, to generate the F_1_ generation. Unrelated F_1_ chickens were selected to breed the F_2_ generation: 25 males × 406 females from WL/DX parental and 24 males × 233 females from DX/WL parental. A total of 1893 F_2_ hens were generated. Detailed information about the resource population can be found in previously published studies^35,36^.

### Phenotypic trait

Hens were euthanized with 60–70% carbon dioxide gas at 72 weeks of age. A total of 1456 hens were used to record the different follicle numbers, including number of pre-ovulatory hierarchical follicles (POF) (>8 mm in diameter), number of small yellow follicles (SYF) (5–8 mm), and number of atresia follicles (AF). If the initial phenotypic data were not normally distributed, they were transformed by using box-cox method in the JMP software to obtain a normal distribution for all subsequent analyses. Means, standard errors, fixed effects, and covariates were calculated using the JMP statistical software^37^. Heritability was estimated by ASReml^38^.

### DNA isolation, genotyping, quality control, and imputation

Blood was collected from the wing vein and genomic DNA was isolated using the standard phenol/chloroform method. Genotyping was performed on 1456 F_2_ hens, using the 600 K Affymetrix Axiom Chicken Genotyping Array (Affymetrix, Inc., USA). Quality control of genotyping data was initially conducted using the Affymetrix Power Tools v1.16.0 (APT) (http://affymetrix.com/) software with dish quality control >0.82, sample call rate >97%, and SNP call rate ≥95% for the downstream analyses. Further quality control and genotyping were implemented by the PLINK v1.90 software^39^ with minor allele frequency (MAF) ≥5% and Hardy-Weinberg equilibrium (HWE) test P ≥ 1 × e10^−6^. Before the GWA analysis, an imputation was performed using the BEAGLE v4.0 procedure^40^ to avoid some sporadic missing genotypes. Besides, principal component analysis and independent inspection number was implemented in the PLINK package to determine the thresholds for genome-wide significant and suggestive associations. Finally, a total of 59,286 independent tests were obtained. The cutoff threshold of genome-wide significant and suggestive P-values were 8.43 × 10^−7^ (0.05/59,286) and 1.69 × 10^−5^ (1.00/59,286), respectively.

### Statistical analyses

The SNPs significantly associated with the respective phenotypic traits were identified using both genome-wide efficient mixed-model association (GEMMA)^41^ and the Bayesian methods in Gensel software^42^. The GEMMA model can be written as

1 $$y=W\alpha +x\beta +\mu +\varepsilon $$

where *y* represents a vector of phenotypic values for *n* individuals; *W* is a matrix of covariates (fixed effects with a column vector of 1 and top five PCs), *α* is a vector of the corresponding coefficients including the intercept; *x* is a vector of the genotypes of the SNP marker, *β* is the effect size of the marker; *μ* is a vector of random individual effects; *ε* is a vector of random error. Bayesian methods can be used to genomic prediction of breeding values and GWAS^43–45^.

The Bayes B model was described as

2 $$y=xb+\sum _{j}^{k}\,{z}_{j}\,{\alpha }_{j}\,{\delta }_{j}+\varepsilon $$

where *y* is a vector of phenotypes; *x* is an incidence matrix to account for fixed effects on phenotypes; *b* is a vector of fixed effects; *z*_*j*_ is a vector of genotypes covariates at SNP *j* (AA = −10, AB = 0, and BB =  + 10); *α*_*j*_ is the allele substitution effect of SNP *j*; *δ*_*j*_ is a parameter that indicates whether SNP *j* was included in the Markov chain Monte Carlo (MCMC) chain; *ε* is the error associated with the analysis.

Manhattan plots and quantile-quantile (QQ) plots of each trait were obtained by the “gap” package^46,47^ in R project. The GenABEL package was used to calculate the genomic inflation factor *λ*. Linkage disequilibrium (LD) analysis was determined by Haploview v4.2^48^ and assessed by D′ ≥0.8 and r^2^ ≥1/3 indicating strong LD status. The contributions to phenotypic variance (CPV) of the significant SNPs were calculated in GCTA based on the genetic relationship matrix (GRM) built by PLINK.

### Candidate genes

Variant Effect Predictor (VEP) and BioMart tools were performed to identify the candidate genes that located the significant SNPs^49,50^. A mutual information network was generated based on current results using minet package for R^50^ with the Aracne algorithm. The network graphs were exported from R for visualization in the MCODE package within Cytoscape 3.2.1^52,53^. Using this visualization tool, we explored networks where one or more SNP was connected via genes.

## Electronic supplementary material

Supplementary Information
