# Supplementary material for: Genetic Architecture and Candidate Genes Identified for Follicle Number in Chicken
Source: Sci Rep. 2017 Nov 27;7:16412. doi: 10.1038/s41598-017-16557-1 (PMC5703906; doi:10.1038/s41598-017-16557-1)
Supplement: Supplementary file 1 — Supplementary Information [file 41598_2017_16557_MOESM1_ESM.doc]

SUPPLEMENTRAY INFORMATION

Genetic Architecture and Candidate Genes Identified for Follicle Number in Chicken

Manman Shen^12¶^, Hongyan Sun^2¶^, Liang Qu^1^, Meng Ma^1^, Taocun Dou^1^, Jian Lu^1^, Jun Guo^1^, Yuping Hu^1^, Xingguo Wang^1^, Yongfeng Li^1^, Kehua Wang^1*^, Ning Yang^3*^

^1^Jiangsu Institute of Poultry Science, Chinese Academy of Agricultural Science, Yangzhou, China

^2^ College of Animal Science and Technology, Yangzhou University, Yangzhou, China

^3^National Engineering Laboratory for Animal Breeding and MOA Key Laboratory of Animal Genetics and Breeding, College of Animal Science and Technology, China Agricultural University, Beijing, China

^*^Corresponding author

E-mail: [sqbreeding@126.com (KH](mailto:sqbreeding@126.com(KH)W)

nyang@cau.edu.cn(NY)

^¶^Equal contributors.

**Table S1** Summary of genetic analysis of different types of follicular

| Trait | POF | SYF | AF |
| --- | --- | --- | --- |
| POF | 0.13 | 0.46 | 0.35 |
| SYF | 0.12 | 0.28 | 0.14 |
| AF | 0.02 | 0.12 | 0.05 |

Diagonal, heritability estimates. Lower triangle, phenotypic correlations. Upper triangle: genetic correlations.

**Table S2** Genome-wide and suggestive significant SNPs associated with SYF via univariate analyses in GEMMA

| Chr | SNP | Position | Minor/  Major allele | MAF | beta | se | l_remle | l_mle | p_wald | p_lrt | p_score |
| --- | --- | --- | --- | --- | --- | --- | --- | --- | --- | --- | --- |
| 28 | rs317889060 | 2012197 | C/T | 0.231 | 3.78E-01 | 5.17E-02 | 9.33E-01 | 8.49E-01 | 4.46E-13 | 5.05E-13 | 2.65E-12 |
| 28 | rs316038837 | 1947008 | T/C | 0.176 | 4.02E-01 | 5.79E-02 | 9.36E-01 | 8.49E-01 | 5.78E-12 | 6.57E-12 | 2.87E-11 |
| 28 | rs16210881 | 1829370 | G/A | 0.174 | 3.62E-01 | 5.53E-02 | 9.59E-01 | 8.73E-01 | 8.75E-11 | 9.27E-11 | 3.09E-10 |
| 28 | rs313648246 | 2010929 | A/T | 0.315 | 2.90E-01 | 4.52E-02 | 9.51E-01 | 8.63E-01 | 1.84E-10 | 2.01E-10 | 6.73E-10 |
| 28 | rs317537249 | 1843976 | T/C | 0.277 | 2.84E-01 | 4.62E-02 | 9.62E-01 | 8.74E-01 | 1.02E-09 | 1.08E-09 | 3.14E-09 |
| 28 | rs313040427 | 1712749 | T/C | 0.167 | 3.49E-01 | 5.73E-02 | 9.95E-01 | 9.08E-01 | 1.40E-09 | 1.36E-09 | 3.36E-09 |
| 28 | rs314802132 | 1542976 | C/A | 0.208 | 3.09E-01 | 5.08E-02 | 9.96E-01 | 9.10E-01 | 1.57E-09 | 1.53E-09 | 3.75E-09 |
| 28 | rs312307881 | 1619315 | T/C | 0.19 | 3.24E-01 | 5.36E-02 | 1.00E+00 | 9.15E-01 | 1.79E-09 | 1.72E-09 | 4.10E-09 |
| 28 | rs316676992 | 1849612 | A/G | 0.276 | 2.79E-01 | 4.62E-02 | 9.66E-01 | 8.77E-01 | 2.07E-09 | 2.18E-09 | 6.03E-09 |
| 28 | rs316754909 | 1944255 | T/C | 0.259 | 2.90E-01 | 4.81E-02 | 9.68E-01 | 8.78E-01 | 2.25E-09 | 2.35E-09 | 6.40E-09 |
| 28 | rs314330209 | 1990610 | G/A | 0.276 | 2.81E-01 | 4.65E-02 | 9.38E-01 | 8.49E-01 | 2.06E-09 | 2.38E-09 | 7.59E-09 |
| 28 | rs314735191 | 1514399 | T/C | 0.188 | 3.18E-01 | 5.36E-02 | 1.02E+00 | 9.35E-01 | 3.49E-09 | 3.22E-09 | 6.88E-09 |
| 28 | rs316105069 | 2123244 | A/G | 0.137 | 3.66E-01 | 6.19E-02 | 9.96E-01 | 9.08E-01 | 4.40E-09 | 4.28E-09 | 9.99E-09 |
| 28 | rs317641108 | 1926277 | A/G | 0.24 | 2.95E-01 | 5.02E-02 | 1.03E+00 | 9.44E-01 | 4.99E-09 | 4.54E-09 | 9.31E-09 |
| 28 | rs318236639 | 1929393 | A/G | 0.245 | 2.91E-01 | 4.96E-02 | 1.04E+00 | 9.50E-01 | 5.32E-09 | 4.80E-09 | 9.61E-09 |
| 28 | rs315715629 | 1761595 | T/C | 0.245 | 2.93E-01 | 4.99E-02 | 1.04E+00 | 9.55E-01 | 5.70E-09 | 5.10E-09 | 1.00E-08 |
| 28 | rs313479236 | 1821752 | C/T | 0.228 | 2.99E-01 | 5.16E-02 | 1.07E+00 | 9.79E-01 | 8.23E-09 | 7.16E-09 | 1.29E-08 |
| 28 | rs15246876 | 2022043 | C/T | 0.3 | 2.65E-01 | 4.57E-02 | 9.76E-01 | 8.87E-01 | 8.27E-09 | 8.49E-09 | 2.11E-08 |
| 28 | rs316444293 | 2342019 | C/A | 0.162 | 3.29E-01 | 5.77E-02 | 9.93E-01 | 9.04E-01 | 1.41E-08 | 1.39E-08 | 3.11E-08 |
| 28 | rs315788897 | 1699155 | T/C | 0.145 | 3.45E-01 | 6.09E-02 | 1.07E+00 | 9.77E-01 | 1.75E-08 | 1.53E-08 | 2.66E-08 |
| 28 | rs315697880 | 1623888 | C/T | 0.162 | 3.28E-01 | 5.78E-02 | 1.02E+00 | 9.36E-01 | 1.70E-08 | 1.57E-08 | 3.13E-08 |
| 17 | rs312873273 | 1366852 | G/C | 0.213 | 3.02E-01 | 5.33E-02 | 9.22E-01 | 8.30E-01 | 1.77E-08 | 2.20E-08 | 6.94E-08 |
| 28 | rs313972624 | 1732609 | C/A | 0.243 | 2.80E-01 | 5.00E-02 | 1.03E+00 | 9.47E-01 | 2.42E-08 | 2.22E-08 | 4.22E-08 |
| 28 | rs314385559 | 2367109 | A/G | 0.165 | 3.19E-01 | 5.75E-02 | 9.83E-01 | 8.94E-01 | 3.35E-08 | 3.40E-08 | 7.72E-08 |
| 28 | rs317337799 | 1754154 | C/T | 0.328 | 2.51E-01 | 4.55E-02 | 1.03E+00 | 9.38E-01 | 3.93E-08 | 3.63E-08 | 6.92E-08 |
| 28 | rs313040269 | 1985086 | C/T | 0.395 | 2.40E-01 | 4.36E-02 | 1.05E+00 | 9.53E-01 | 4.09E-08 | 3.64E-08 | 6.52E-08 |
| 28 | rs316358363 | 1626264 | A/G | 0.162 | 3.15E-01 | 5.77E-02 | 1.03E+00 | 9.39E-01 | 5.87E-08 | 5.45E-08 | 1.02E-07 |
| 28 | rs14306530 | 1499327 | T/C | 0.15 | 3.24E-01 | 5.97E-02 | 1.08E+00 | 9.93E-01 | 6.60E-08 | 5.73E-08 | 9.07E-08 |
| 28 | rs16211213 | 1541935 | A/G | 0.162 | 3.15E-01 | 5.81E-02 | 1.05E+00 | 9.67E-01 | 7.14E-08 | 6.36E-08 | 1.08E-07 |
| 28 | rs312587681 | 2335214 | T/C | 0.16 | 3.18E-01 | 5.87E-02 | 1.01E+00 | 9.21E-01 | 6.78E-08 | 6.48E-08 | 1.28E-07 |
| 28 | rs314643774 | 1910877 | G/A | 0.323 | 2.46E-01 | 4.55E-02 | 1.03E+00 | 9.40E-01 | 7.63E-08 | 7.03E-08 | 1.30E-07 |
| 28 | rs312747975 | 1247445 | C/T | 0.257 | 2.60E-01 | 4.84E-02 | 1.07E+00 | 9.80E-01 | 8.61E-08 | 7.55E-08 | 1.22E-07 |
| 28 | rs15246822 | 2009905 | A/G | 0.24 | 2.60E-01 | 4.83E-02 | 1.02E+00 | 9.31E-01 | 8.72E-08 | 8.15E-08 | 1.54E-07 |
| 28 | rs313649151 | 1248415 | T/C | 0.219 | 2.68E-01 | 5.01E-02 | 1.07E+00 | 9.84E-01 | 1.03E-07 | 9.00E-08 | 1.43E-07 |
| 28 | rs16211139 | 1602011 | G/C | 0.24 | 2.69E-01 | 5.05E-02 | 1.08E+00 | 9.89E-01 | 1.17E-07 | 1.02E-07 | 1.60E-07 |
| 28 | rs316220133 | 1964341 | G/A | 0.225 | 2.68E-01 | 5.06E-02 | 1.03E+00 | 9.36E-01 | 1.37E-07 | 1.27E-07 | 2.30E-07 |
| 28 | rs316907030 | 1749175 | G/A | 0.322 | 2.41E-01 | 4.58E-02 | 1.03E+00 | 9.39E-01 | 1.56E-07 | 1.45E-07 | 2.62E-07 |
| 28 | rs317996619 | 2017247 | T/C | 0.274 | 2.46E-01 | 4.69E-02 | 1.03E+00 | 9.39E-01 | 1.85E-07 | 1.71E-07 | 3.05E-07 |
| 28 | rs14305824 | 2179908 | A/G | 0.178 | 2.87E-01 | 5.49E-02 | 1.07E+00 | 9.78E-01 | 1.95E-07 | 1.73E-07 | 2.74E-07 |
| 28 | rs316367068 | 1524526 | A/G | 0.216 | 2.71E-01 | 5.19E-02 | 1.05E+00 | 9.60E-01 | 1.94E-07 | 1.76E-07 | 2.95E-07 |
| 28 | rs312811524 | 1786822 | C/G | 0.293 | 2.38E-01 | 4.55E-02 | 1.02E+00 | 9.35E-01 | 1.89E-07 | 1.76E-07 | 3.19E-07 |
| 28 | rs316413369 | 1537133 | T/C | 0.279 | 2.40E-01 | 4.56E-02 | 9.82E-01 | 8.93E-01 | 1.71E-07 | 1.76E-07 | 3.81E-07 |
| 28 | rs13663720 | 1788269 | T/C | 0.157 | 3.19E-01 | 6.12E-02 | 1.09E+00 | 1.00E+00 | 2.13E-07 | 1.85E-07 | 2.72E-07 |
| 28 | rs314187546 | 1444284 | A/G | 0.21 | 2.66E-01 | 5.11E-02 | 1.07E+00 | 9.82E-01 | 2.16E-07 | 1.91E-07 | 2.98E-07 |
| 28 | rs312586874 | 1449431 | A/G | 0.224 | 2.65E-01 | 5.10E-02 | 1.10E+00 | 1.01E+00 | 2.26E-07 | 1.96E-07 | 2.86E-07 |
| 28 | rs316945505 | 2388737 | T/G | 0.156 | 3.01E-01 | 5.78E-02 | 1.07E+00 | 9.79E-01 | 2.25E-07 | 1.98E-07 | 3.11E-07 |
| 28 | rs313136262 | 2391682 | T/G | 0.155 | 3.02E-01 | 5.84E-02 | 1.06E+00 | 9.76E-01 | 2.60E-07 | 2.30E-07 | 3.62E-07 |
| 17 | rs15803483 | 2391488 | A/G | 0.098 | 2.61E-01 | 7.34E-02 | 1.08E+00 | 9.84E-01 | 3.89E-07 | 3.49E-07 | 4.40E-07 |
| 28 | rs317962183 | 2017285 | T/C | 0.261 | 2.38E-01 | 4.72E-02 | 1.06E+00 | 9.69E-01 | 5.16E-07 | 4.60E-07 | 7.20E-07 |
| 28 | rs16211274 | 1463378 | A/G | 0.254 | 2.46E-01 | 4.89E-02 | 1.08E+00 | 9.93E-01 | 5.35E-07 | 4.70E-07 | 6.94E-07 |
| 28 | rs318099911 | 2163700 | C/T | 0.145 | 3.06E-01 | 6.09E-02 | 1.10E+00 | 1.02E+00 | 5.74E-07 | 4.98E-07 | 6.95E-07 |
| 28 | rs316574537 | 2432874 | T/C | 0.167 | 2.86E-01 | 5.68E-02 | 1.05E+00 | 9.60E-01 | 5.49E-07 | 4.98E-07 | 8.03E-07 |
| 17 | rs316071981 | 2388277 | G/A | 0.277 | 2.02E-01 | 5.01E-02 | 1.09E+00 | 9.92E-01 | 5.93E-07 | 5.24E-07 | 6.71E-07 |
| 28 | rs314231916 | 1709862 | A/G | 0.318 | 2.29E-01 | 4.56E-02 | 1.03E+00 | 9.40E-01 | 5.92E-07 | 5.52E-07 | 9.52E-07 |
| 28 | rs313608400 | 1921039 | A/G | 0.189 | 2.70E-01 | 5.42E-02 | 1.12E+00 | 1.03E+00 | 6.75E-07 | 5.86E-07 | 7.91E-07 |
| 28 | rs315973958 | 2308710 | C/T | 0.12 | 3.36E-01 | 6.75E-02 | 1.07E+00 | 9.75E-01 | 7.50E-07 | 6.65E-07 | 1.01E-06 |
| 28 | rs314907214 | 1443055 | G/A | 0.235 | 2.49E-01 | 5.02E-02 | 1.09E+00 | 1.00E+00 | 7.98E-07 | 6.98E-07 | 9.92E-07 |
| 28 | rs14305841 | 2185417 | T/C | 0.135 | 3.11E-01 | 6.29E-02 | 1.13E+00 | 1.04E+00 | 8.64E-07 | 7.51E-07 | 9.94E-07 |
| 28 | rs13545894 | 1871024 | T/C | 0.313 | 2.27E-01 | 4.62E-02 | 1.04E+00 | 9.54E-01 | 9.88E-07 | 9.03E-07 | 1.46E-06 |
| 28 | rs315675468 | 1899444 | A/G | 0.312 | 2.26E-01 | 4.62E-02 | 1.04E+00 | 9.56E-01 | 1.08E-06 | 9.85E-07 | 1.57E-06 |
| 28 | rs314625273 | 2230957 | G/C | 0.13 | 3.14E-01 | 6.42E-02 | 1.15E+00 | 1.06E+00 | 1.14E-06 | 9.99E-07 | 1.27E-06 |
| 28 | rs313767061 | 1946454 | G/A | 0.399 | 1.99E-01 | 4.07E-02 | 1.02E+00 | 9.32E-01 | 1.14E-06 | 1.07E-06 | 1.84E-06 |
| 28 | rs16210121 | 1901935 | G/A | 0.301 | 2.29E-01 | 4.70E-02 | 1.07E+00 | 9.77E-01 | 1.24E-06 | 1.10E-06 | 1.64E-06 |
| 28 | rs13546017 | 2257585 | C/T | 0.325 | 2.25E-01 | 4.62E-02 | 1.09E+00 | 1.00E+00 | 1.31E-06 | 1.15E-06 | 1.61E-06 |
| 28 | rs318148179 | 2383692 | T/C | 0.155 | 2.90E-01 | 5.98E-02 | 1.07E+00 | 9.76E-01 | 1.36E-06 | 1.21E-06 | 1.80E-06 |
| 28 | rs14305734 | 1987232 | G/A | 0.373 | 2.10E-01 | 4.36E-02 | 1.07E+00 | 9.74E-01 | 1.61E-06 | 1.42E-06 | 2.10E-06 |
| 28 | rs316768215 | 1423922 | C/T | 0.271 | 2.32E-01 | 4.82E-02 | 1.11E+00 | 1.03E+00 | 1.65E-06 | 1.44E-06 | 1.91E-06 |
| 28 | rs317956782 | 2345287 | A/G | 0.154 | 2.92E-01 | 6.03E-02 | 1.01E+00 | 9.16E-01 | 1.49E-06 | 1.46E-06 | 2.64E-06 |
| 28 | rs316511155 | 1457474 | C/T | 0.332 | 2.16E-01 | 4.49E-02 | 1.04E+00 | 9.50E-01 | 1.60E-06 | 1.47E-06 | 2.38E-06 |
| 28 | rs317428299 | 1965318 | A/G | 0.466 | 2.09E-01 | 4.36E-02 | 1.07E+00 | 9.75E-01 | 1.74E-06 | 1.54E-06 | 2.27E-06 |
| 28 | rs312297279 | 1963387 | T/C | 0.465 | 2.09E-01 | 4.37E-02 | 1.07E+00 | 9.75E-01 | 1.82E-06 | 1.61E-06 | 2.37E-06 |
| 28 | rs316129460 | 1294715 | G/C | 0.173 | 2.65E-01 | 5.53E-02 | 1.12E+00 | 1.03E+00 | 1.91E-06 | 1.66E-06 | 2.18E-06 |
| 28 | rs314550587 | 1615475 | T/C | 0.327 | 2.18E-01 | 4.55E-02 | 1.02E+00 | 9.35E-01 | 1.81E-06 | 1.71E-06 | 2.91E-06 |
| 28 | rs314283438 | 1620554 | T/C | 0.327 | 2.18E-01 | 4.55E-02 | 1.02E+00 | 9.35E-01 | 1.81E-06 | 1.71E-06 | 2.91E-06 |
| 28 | rs316436889 | 1475894 | G/T | 0.333 | 2.13E-01 | 4.50E-02 | 1.03E+00 | 9.45E-01 | 2.46E-06 | 2.29E-06 | 3.70E-06 |
| 28 | rs316976813 | 1622196 | C/T | 0.327 | 2.15E-01 | 4.55E-02 | 1.02E+00 | 9.35E-01 | 2.46E-06 | 2.33E-06 | 3.91E-06 |
| 28 | rs317609954 | 1811768 | A/G | 0.257 | 2.30E-01 | 4.88E-02 | 1.06E+00 | 9.72E-01 | 2.65E-06 | 2.37E-06 | 3.48E-06 |
| 28 | rs317858034 | 2042162 | C/T | 0.215 | 2.52E-01 | 5.40E-02 | 1.08E+00 | 9.87E-01 | 3.37E-06 | 2.99E-06 | 4.21E-06 |
| 28 | rs317955117 | 2139848 | G/C | 0.195 | 2.60E-01 | 5.60E-02 | 1.07E+00 | 9.84E-01 | 3.72E-06 | 3.31E-06 | 4.68E-06 |
| 28 | rs318148832 | 1669231 | A/G | 0.304 | 2.16E-01 | 4.66E-02 | 1.06E+00 | 9.70E-01 | 3.72E-06 | 3.35E-06 | 4.94E-06 |
| 7 | rs316846127 | 2.7E+07 | A/G | 0.207 | -2.46E-01 | 5.32E-02 | 1.10E+00 | 1.01E+00 | 3.92E-06 | 3.42E-06 | 4.53E-06 |
| 4 | rs316614471 | 6.9E+07 | G/A | 0.495 | 2.17E-01 | 4.74E-02 | 1.10E+00 | 1.00E+00 | 5.29E-06 | 4.59E-06 | 6.12E-06 |
| 28 | rs317040063 | 2434037 | A/G | 0.185 | 2.54E-01 | 5.54E-02 | 1.03E+00 | 9.46E-01 | 5.11E-06 | 4.78E-06 | 7.50E-06 |
| 28 | rs315733874 | 1476734 | A/G | 0.331 | 2.05E-01 | 4.52E-02 | 1.03E+00 | 9.42E-01 | 6.03E-06 | 5.66E-06 | 9.04E-06 |
| 28 | rs316957526 | 1962597 | A/G | 0.455 | 1.98E-01 | 4.37E-02 | 1.08E+00 | 9.83E-01 | 6.59E-06 | 5.81E-06 | 8.07E-06 |
| 28 | rs316082769 | 1491331 | G/T | 0.223 | 2.28E-01 | 5.03E-02 | 1.06E+00 | 9.69E-01 | 6.60E-06 | 5.94E-06 | 8.59E-06 |
| 28 | rs317516380 | 1534675 | A/C | 0.224 | 2.25E-01 | 5.01E-02 | 1.06E+00 | 9.64E-01 | 7.39E-06 | 6.70E-06 | 9.83E-06 |
| 28 | rs13546047 | 2434750 | T/C | 0.175 | 2.56E-01 | 5.71E-02 | 1.05E+00 | 9.59E-01 | 7.66E-06 | 7.02E-06 | 1.04E-05 |
| 28 | rs314863602 | 1129997 | C/A | 0.225 | 2.19E-01 | 4.93E-02 | 1.17E+00 | 1.08E+00 | 9.17E-06 | 8.26E-06 | 9.67E-06 |
| 4 | rs317669292 | 6.8E+07 | A/C | 0.482 | 2.37E-01 | 5.35E-02 | 1.09E+00 | 9.95E-01 | 9.83E-06 | 8.56E-06 | 1.14E-05 |
| 28 | rs14305648 | 1876103 | C/T | 0.283 | 2.07E-01 | 4.67E-02 | 1.08E+00 | 9.92E-01 | 9.95E-06 | 8.82E-06 | 1.19E-05 |
| 28 | rs15249505 | 1444458 | G/A | 0.346 | 1.98E-01 | 4.47E-02 | 1.08E+00 | 9.91E-01 | 1.04E-05 | 9.19E-06 | 1.25E-05 |
| 17 | rs313151732 | 8421153 | T/C | 0.278 | 2.17E-01 | 4.92E-02 | 1.11E+00 | 1.02E+00 | 1.06E-05 | 9.33E-06 | 1.19E-05 |
| 17 | rs14097203 | 9481782 | T/G | 0.201 | 2.36E-01 | 5.34E-02 | 1.14E+00 | 1.05E+00 | 1.09E-05 | 9.64E-06 | 1.16E-05 |
| 17 | rs315680387 | 8805635 | G/T | 0.313 | 2.03E-01 | 4.61E-02 | 1.13E+00 | 1.03E+00 | 1.16E-05 | 1.02E-05 | 1.25E-05 |
| 17 | rs315702635 | 8232439 | A/G | 0.186 | 2.39E-01 | 5.44E-02 | 1.11E+00 | 1.02E+00 | 1.23E-05 | 1.08E-05 | 1.35E-05 |
| 17 | rs312844291 | 8260295 | A/G | 0.236 | -2.27E-01 | 5.18E-02 | 1.06E+00 | 9.66E-01 | 1.24E-05 | 1.11E-05 | 1.58E-05 |
| 17 | rs314977935 | 8653471 | G/A | 0.161 | 2.54E-01 | 5.84E-02 | 1.09E+00 | 9.98E-01 | 1.41E-05 | 1.25E-05 | 1.65E-05 |
| 17 | rs316673254 | 6756072 | G/A | 0.399 | 1.89E-01 | 4.36E-02 | 1.11E+00 | 1.01E+00 | 1.50E-05 | 1.32E-05 | 1.67E-05 |
| 17 | rs314387672 | 496742 | C/T | 0.331 | -1.83E-01 | 4.23E-02 | 1.11E+00 | 1.02E+00 | 1.55E-05 | 1.36E-05 | 1.70E-05 |
| 17 | rs313755967 | 6492686 | A/G | 0.161 | 2.51E-01 | 5.79E-02 | 1.05E+00 | 9.64E-01 | 1.52E-05 | 1.40E-05 | 2.02E-05 |
| 17 | rs15790486 | 8804849 | C/T | 0.148 | 2.60E-01 | 6.01E-02 | 1.06E+00 | 9.72E-01 | 1.56E-05 | 1.42E-05 | 1.99E-05 |
| 1 | rs313777254 | 1.9E+08 | G/C | 0.435 | 1.81E-01 | 4.19E-02 | 1.16E+00 | 1.07E+00 | 1.71E-05 | 1.53E-05 | 1.78E-05 |
| 28 | rs15249538 | 1441496 | A/G | 0.342 | 1.92E-01 | 4.46E-02 | 1.09E+00 | 9.97E-01 | 1.82E-05 | 1.61E-05 | 2.13E-05 |
| 4 | rs15611747 | 6.8E+07 | C/T | 0.476 | 2.29E-01 | 5.33E-02 | 1.08E+00 | 9.86E-01 | 1.86E-05 | 1.63E-05 | 2.19E-05 |
| 28 | rs316642867 | 1125654 | A/G | 0.209 | -2.27E-01 | 5.30E-02 | 1.08E+00 | 9.87E-01 | 1.93E-05 | 1.71E-05 | 2.29E-05 |
| 28 | rs312509372 | 2019548 | A/G | 0.221 | 2.16E-01 | 5.05E-02 | 1.11E+00 | 1.02E+00 | 1.99E-05 | 1.74E-05 | 2.16E-05 |
| 28 | rs314129973 | 2458154 | T/C | 0.223 | 2.23E-01 | 5.22E-02 | 1.10E+00 | 1.01E+00 | 2.12E-05 | 1.87E-05 | 2.37E-05 |
| 17 | rs314478835 | 8385013 | A/G | 0.096 | 2.45E-01 | 7.42E-02 | 1.09E+00 | 9.91E-01 | 9.90E-05 | 8.87E-05 | 1.09E-05 |
| 17 | rs315972958 | 8389468 | A/G | 0.096 | 2.45E-01 | 7.42E-02 | 1.09E+00 | 9.91E-01 | 9.90E-05 | 8.87E-05 | 1.09E-05 |

**Table S3** Significant SNPs on GGA1 association with POF

| Chr | SNP | Gene | Location | Position | Minor/  Major allele | MAF | beta | se | l_remle | l_mle | p_wald | p_lrt | p_score |
| --- | --- | --- | --- | --- | --- | --- | --- | --- | --- | --- | --- | --- | --- |
| 1 | rs14920355 | DCLK1 | Intron variant | 172084219 | C/T | 0.246 | -2.34E-01 | 5.12E-02 | 3.28E-01 | 2.64E-01 | 5.26E-07 | 4.56E-07 | 9.00E-07 |
| 1 | rs316013853 | NBEA | intron variant | 172453837 | C/T | 0.278 | -2.16E-01 | 4.80E-02 | 3.35E-01 | 2.72E-01 | 7.59E-07 | 6.47E-07 | 1.18E-06 |
| 1 | rs317447068 | DCLK1 | intron variant | 172200118 | T/C | 0.281 | -2.15E-01 | 4.86E-02 | 3.32E-01 | 2.68E-01 | 1.04E-07 | 7.91E-07 | 1.66E-06 |
| 1 | rs314080199 | U6kb/  SMAD9 | intergenic variant | 171748999 | T/C | 0.311 | -2.06E-01 | 4.69E-02 | 3.15E-01 | 2.51E-01 | 1.15E-07 | 1.05E-07 | 2.32E-07 |

**Table S4** The highest effect of SNPs on the SYF trait

| Chr | SNP | Position | Window(SNP) | %GV | Model freq. |
| --- | --- | --- | --- | --- | --- |
| 28 | rs13663720 | 1788269 | 312 | 0.66 | 0.0049 |
| 28 | rs14305824 | 2179908 | 263 | 0.65 | 0.0063 |
| 28 | rs14306530 | 1499327 | 188 | 0.64 | 0.0053 |
| 28 | rs16210881 | 1829370 | 322 | 0.73 | 0.0042 |
| 28 | rs16211139 | 1602011 | 163 | 0.75 | 0.0053 |
| 28 | rs16211213 | 1541935 | 322 | 0.66 | 0.0039 |
| 28 | rs16211274 | 1463378 | 179 | 1.26 | 0.0046 |
| 28 | rs312586874 | 1449431 | 215 | 0.56 | 0.0053 |
| 28 | rs312811524 | 1786822 | 128 | 0.74 | 0.0043 |
| 17 | rs312873273 | 1366852 | 275 | 0.55 | 0.0042 |
| 28 | rs313479236 | 1821752 | 129 | 0.58 | 0.0049 |
| 28 | rs313648246 | 2010929 | 255 | 0.57 | 0.0053 |
| 28 | rs313972624 | 1732609 | 216 | 0.78 | 0.0068 |
| 28 | rs314231916 | 1709862 | 316 | 0.69 | 0.0068 |
| 28 | rs314643774 | 1910877 | 255 | 0.76 | 0.0033 |
| 28 | rs314802132 | 1542976 | 218 | 0.54 | 0.0053 |
| 28 | rs314907214 | 1443055 | 326 | 0.53 | 0.0125 |
| 28 | rs315697880 | 1623888 | 155 | 0.53 | 0.0048 |
| 28 | rs316038837 | 1947008 | 148 | 0.67 | 0.0063 |
| 28 | rs316105069 | 2123244 | 156 | 0.69 | 0.0037 |
| 28 | rs316358363 | 1626264 | 175 | 0.55 | 0.0052 |
| 28 | rs316367068 | 1524526 | 145 | 0.63 | 0.0038 |
| 28 | rs316413369 | 1537133 | 279 | 0.66 | 0.0056 |
| 28 | rs316444293 | 2342019 | 125 | 0.63 | 0.0048 |
| 28 | rs316574537 | 2432874 | 219 | 0.52 | 0.0059 |
| 28 | rs317337799 | 1754154 | 226 | 0.53 | 0.0145 |
| 28 | rs318099911 | 2163700 | 268 | 0.54 | 0.0053 |
| 28 | rs318236639 | 1929393 | 169 | 1.23 | 0.0056 |
| 17 | rs316071981 | 2388277 | 202 | 0.63 | 0.0037 |
| 2 | rs312594368 | 142427903 | 158 | 0.84 | 0.0049 |
| 28 | rs315908028 | 1302530 | 187 | 1.01 | 0.0059 |
| 28 | rs15249538 | 1441496 | 201 | 1.35 | 0.0082 |
| 28 | rs317610409 | 1584842 | 149 | 1.63 | 0.0046 |
| 28 | rs313972672 | 1662739 | 79 | 0.92 | 0.0038 |
| 28 | rs314382657 | 1665602 | 187 | 1.2 | 0.0028 |
| 28 | rs313706329 | 1868848 | 145 | 2.1 | 0.0047 |
| 28 | rs314592481 | 2002372 | 125 | 0.69 | 0.0052 |
| 28 | rs315162441 | 2153730 | 169 | 1.37 | 0.0062 |
| 28 | rs316485971 | 2317176 | 182 | 0.69 | 0.0029 |
| 28 | rs16703946 | 1301624 | 157 | 0.82 | 0.0036 |
| 28 | rs316998684 | 2225605 | 166 | 1.26 | 0.0027 |
| 28 | rs312395961 | 2400026 | 149 | 0.64 | 0.0067 |
